# Adaptive delivery of continuous and delayed feedback deep brain stimulation - a computational study

**DOI:** 10.1038/s41598-019-47036-4

**Published:** 2019-07-22

**Authors:** Oleksandr V. Popovych, Peter A. Tass

**Affiliations:** 1 Institute of Neuroscience and Medicine - Brain & Behaviour (INM-7), Research Centre Juelich, Juelich, Germany; 2 Department of Neurosurgery, Stanford University, Stanford, California United States

**Keywords:** Computational science, Dynamical systems, Parkinson's disease

## Abstract

Adaptive deep brain stimulation (aDBS) is a closed-loop method, where high-frequency DBS is turned on and off according to a feedback signal, whereas conventional high-frequency DBS (cDBS) is delivered permanently. Using a computational model of subthalamic nucleus and external globus pallidus, we extend the concept of adaptive stimulation by adaptively controlling not only continuous, but also demand-controlled stimulation. Apart from aDBS and cDBS, we consider continuous pulsatile linear delayed feedback stimulation (cpLDF), specifically designed to induce desynchronization. Additionally, we combine adaptive on-off delivery with continuous delayed feedback modulation by introducing adaptive pulsatile linear delayed feedback stimulation (apLDF), where cpLDF is turned on and off using pre-defined amplitude thresholds. By varying the stimulation parameters of cDBS, aDBS, cpLDF, and apLDF we obtain optimal parameter ranges. We reveal a simple relation between the thresholds of the local field potential (LFP) for aDBS and apLDF, the extent of the stimulation-induced desynchronization, and the integral stimulation time required. We find that aDBS and apLDF can be more efficient in suppressing abnormal synchronization than continuous simulation. However, apLDF still remains more efficient and also causes a stronger reduction of the LFP beta burst length. Hence, adaptive on-off delivery may further improve the intrinsically demand-controlled pLDF.

## Introduction

High-frequency (HF) deep brain stimulation (DBS) is the standard therapy for the treatment of essential tremor, dystonia and Parkinson’s disease (PD)^1–4^. To overcome limitations of continuous HF DBS (cDBS), such as side effects, closed-loop and demand-controlled, adaptive DBS (aDBS) was tested in animal and clinical studies^5–20^. For this type of approach, stimulation is aimed to be administered only when necessary and to an extent depending on the measured neuronal activity or symptoms. One of the closed-loop approaches is based on an on-off strategy, where the stimulation is switched on and off when certain events are detected, for example, when a selected biomarker crosses a predefined threshold. Examples for trigger events or biomarkers were action potentials recorded from the primary motor cortex^6^ or the amplitude of the beta-band local field potential (LFP) of the subthalamic nucleus (STN)^7,14–16,18,20^. Interestingly, aDBS could selectively reduce the duration of bursts of the beta-band LFP, where the prevalence of short (long) LFP bursts negatively (positively) correlated with motor impairment off stimulation^19^. By the same token, peripheral signals, reflecting peripheral tremor activity, were used to trigger HF DBS^5,8,9^ or to adapt the amplitude of HF DBS to the amplitude of the ongoing peripheral tremor^17^.

Instead of the on-off strategy discussed above, the stimulation intensity can also be adapted in real time to the amplitude of the biomarker signal^13,17^. To some extent the latter approach mimics closed-loop feedback methods that have been developed in the past for the control of abnormal neuronal synchronization, which is a hallmark of several neurological disorders, like PD^21,22^, essential tremor^23^, epilepsy^24^, and tinnitus^25–27^. For feedback control the mean field, e.g., the LFP of a synchronized population is measured, preprocessed (e.g., filtered, delayed, amplified, etc.) and fed back to the synchronized neuronal population as a stimulation signal^28–38^. Two desynchronizing delayed feedback methods, single- and multi-site linear delayed feedback (LDF) and nonlinear delayed feedback (NDF) were recently adapted and computationally tested for electrical closed-loop DBS^39–41^. Since direct electrical stimulation of the neuronal tissue with smooth and slowly oscillating feedback signals may cause an irreversible charge deposit in the neuronal tissue exceeding safety limits^2,42,43^, the amplitude of the HF train of charge-balanced pulses used by the standard HF DBS is modulated by the slow feedback signal, which constitutes *a pulsatile feedback stimulation* appropriate for electrical DBS^39–41^.

The main goal of this study is to investigate the impact of adaptive on-off delivery on both continuous as well as delayed feedback stimulation. For this, we study differential effects of cDBS and aDBS. Furthermore, to combine adaptive on-off delivery with continuous delayed feedback modulation, we here present a novel method for adaptive brain stimulation technique, adaptive pulsatile LDF (apLDF). To this end, continuous pulsatile LDF (cpLDF)^39–41^ is triggered by the extent of the abnormal neuronal synchrony in an on-off manner. In contrast, so far LDF was not delivered in an adaptive manner. To illustrate the performance of apLDF, we use a physiologically motivated model of interacting populations of STN and external globus pallidus (GPe) neurons suggested for modeling parkinsonian neuronal dynamics^44,45^. The reciprocally connected excitatory (STN) and inhibitory (GPe) neuronal populations may act as a pacemaker, resulting in the emergence of oscillatory activity in PD, as put forward based on pre-clinical studies^46^.

We compare the performance of apLDF, cpLDF, aDBS, and cDBS. It is key to understand the impact of stimulation parameters on the stimulation outcome^47^. Accordingly, in this paper, for all stimulation techniques under study, we systematically vary the stimulation intensity, values of the LFP thresholds used to trigger onsets and offsets of the stimulation as well as the width of the gap between the phases of biphasic charge-balanced asymmetric stimulation pulses. Based on our computational results, aDBS can be at least as effective as cDBS in suppressing abnormal neuronal synchrony. The former can however utilize much less stimulation time such that it becomes much more efficient than cDBS and approaches characteristics of pulsatile LDF, especially, for an interphase gap of moderate width. However, for optimal parameters, apLDF requires less stimulation current in inducing desynchronization than aDBS. We also show that apLDF shortens LFP burst length significantly stronger than cpLDF. Hence, although HF DBS and pulsatile LDF are qualitatively different stimulation techniques, for apLDF vs. cpLDF our computational findings are in agreement with a hypothesis put forward for aDBS vs. cDBS, saying that adaptive stimulation reduces LFP burst length^19^. Furthermore, our computational results show that adaptive on-off delivery may further improve the intrinsically demand-controlled pLDF.

## Methods

### Model

We consider a network of two neuronal populations, which models the dynamics of STN and GPe neurons. Each cell is described by the following system^44^:

1 $${C}_{m}v^{\prime} =-\,{I}_{{\rm{L}}}-{I}_{{\rm{K}}}-{I}_{{\rm{Na}}}-{I}_{{\rm{T}}}-{I}_{{\rm{Ca}}}-{I}_{{\rm{AHP}}}-{I}_{{\rm{syn}}}+{I}_{{\rm{app}}}+{I}_{{\rm{stim}}},$$

2 $$[{\rm{Ca}}]^{\prime} =\varepsilon (-{I}_{{\rm{Ca}}}-{I}_{{\rm{T}}}-{k}_{{\rm{Ca}}}[{\rm{Ca}}]),$$

3 $$X^{\prime} ={\varphi }_{X}({X}_{\infty }(v)-X)/{\tau }_{X}(v).$$

In equations (1)–(3), *v* is a membrane potential of the neuron, the currents *I*_L_, *I*_K_, *I*_Na_, *I*_T_, *I*_Ca_, *I*_AHP_, *I*_syn_, and *I*_app_ are the corresponding leak, potassium, sodium, low threshold calcium, high threshold calcium, afterhyperpolarisation potassium, synaptic, and external current, respectively. [Ca] is the intracellular concentration of Ca^2+^ ions, and *X* = *n*, *h*, *r* are the gating variables.

The following currents from equation (1) attain the same form for both STN and GPe neurons:

$$\begin{array}{ll}{I}_{{\rm{L}}}={g}_{{\rm{L}}}(v-{v}_{L}), & {I}_{{\rm{K}}}={g}_{{\rm{K}}}{n}^{4}(v-{v}_{{\rm{K}}}),\\ {I}_{{\rm{Na}}}={g}_{{\rm{Na}}}{m}_{\infty }^{3}(v)h(v-{v}_{{\rm{Na}}}), & {I}_{{\rm{Ca}}}={g}_{{\rm{Ca}}}{s}_{\infty }^{2}(v)(v-{v}_{{\rm{Ca}}}),\\ {I}_{{\rm{AHP}}}={g}_{{\rm{AHP}}}(v-{v}_{{\rm{K}}})([{\rm{Ca}}]/([{\rm{Ca}}]+{k}_{1})), & \end{array}$$

whereas current *I*_T_ is given by different expressions for the excitatory STN cells and for the inhibitory GPe cells:

$${\rm{STN}}:\,\,{I}_{{\rm{T}}}={g}_{{\rm{T}}}{a}_{\infty }^{3}(v){b}_{\infty }^{2}(r)(v-{v}_{{\rm{Ca}}}),\,{\rm{GPe}}:\,\,{I}_{{\rm{T}}}={g}_{{\rm{T}}}{a}_{\infty }^{3}(v)r(v-{v}_{{\rm{Ca}}}),$$

where ${b}_{\infty }(r)=1/(1+\exp [(r-{\theta }_{b})/{\sigma }_{b}])-1/(1+\exp [\,-\,{\theta }_{b}/{\sigma }_{b}])$. The functions ${X}_{\infty }(v)$ and ${\tau }_{X}(v)$ used in equation (3) and in the above definition of the currents read

$$\begin{array}{ll}{X}_{\infty }(v)=1/(1+\exp [\,-\,(v-{\theta }_{X})/{\sigma }_{X}]), & X=n,h,r,m,s,a,\\ {\tau }_{X}(v)={\tau }_{X}^{0}+{\tau }_{X}^{1}/(1+\exp [\,-\,(v-{\theta }_{X}^{\tau })/{\sigma }_{X}^{\tau }]),\, & X=n,h,r.\end{array}$$

For GPe neurons ${\tau }_{r}(v)={\tau }_{r}$ is a constant parameter.

In our study we consider two interacting populations of *N* = 200 STN and 200 GPe neurons on 1Dim lattices with periodic boundary conditions. Each STN neuron excites a single GPe neuron, whereas each GPe neuron inhibits three neighboring STN neurons, see Supplementary Fig. S1. Microscopic models of this type were introduced and investigated in a number of papers^39,40,44,45,48^, where STN neurons receive an inhibitory input from GPe neurons and, in turn, give an excitatory output to the GPe network. The considered sparse and structured connectivity can support well-pronounced and stable synchronized patterns of the STN-GPe network^44^ as we show below, which will be controlled by an external stimulation. The coupling among the neurons is realized via synaptic currents *I*_syn_ defined in the following way:

$${\rm{STN}}:\,{I}_{{\rm{syn}}}={g}_{{\rm{G}}\to {\rm{S}}}(v-{v}_{{\rm{G}}\to {\rm{S}}})\,\sum \,{s}_{{\rm{j}}},\,{\rm{GPe}}:\,{I}_{{\rm{syn}}}={g}_{{\rm{S}}\to {\rm{G}}}(v-{v}_{{\rm{S}}\to {\rm{G}}})\,\sum \,{s}_{{\rm{j}}},$$

for STN and GPe cells, respectively. *j* is the index of neurons and summations are taken over all presynaptic neurons. The synaptic weights ${g}_{{\rm{S}}\to {\rm{G}}}=0.4\,{\rm{nS}}$/*μ*m^2^ and ${g}_{{\rm{G}}\to {\rm{S}}}=1.38\,{\rm{nS}}$/*μ*m^2^ reflect the strength of the coupling from STN neurons to GPe neurons, and in the opposite direction, respectively. The considered relatively strong GPe-STN coupling reflects the experimental finding reporting the strengthening of the GPe–STN pathway at the dopamine depletion characteristic for PD^49^ and leads to synchronized bursting dynamics of STN neurons. The reversal potentials ${v}_{{\rm{S}}\to {\rm{G}}}=0\,{\rm{mV}}$ and ${v}_{{\rm{G}}\to {\rm{S}}}=-\,100\,{\rm{mV}}$ reflect the excitatory coupling from STN to GPe neurons and inhibitory coupling from GPe to STN, respectively. The equation for the synaptic variables *s*_*j*_ reads:

4 $${s^{\prime} }_{j}=\alpha {H}_{\infty }({v}_{j}-{\theta }_{g})(1-{s}_{j})-\beta {s}_{j},{H}_{\infty }(x)=1/(1+\exp \,[\,-\,(x-{\theta }_{g}^{H})/{\sigma }_{g}^{H}]).$$

We suppose that the neurons in the STN and GPe ensembles are nonidentical. For this, the applied currents ${I}_{{\rm{app}}}={I}_{{\rm{app}},j}$ for STN cells are Gaussian distributed with the mean 10 pA/*μ*m^2^ and the standard deviation 0.015 pA/*μ*m^2^. The parameter $\varepsilon ={\varepsilon }_{j}$ for GPe neurons are also Gaussian distributed with the mean 0.0055 ms^−1^ and the standard deviation $2\cdot {10}^{-5}\,{{\rm{ms}}}^{-1}$. The values of the other parameters for the STN and GPe neurons are listed in Supplementary Table S1.

### Synchronized dynamics of STN neurons

**Figure 1:**
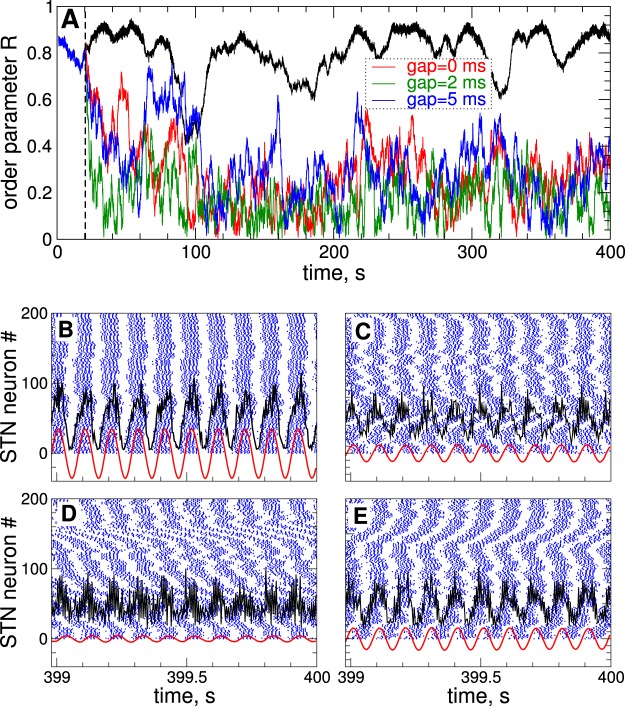
Synchronized dynamics of the STN-GPe neuronal populations (1)–(4) and its suppression by continuous HF DBS. (**A**) Time courses of the order parameter *R* of STN neurons without stimulation (${I}_{{\rm{stim}}}=0$ in equation (1), upper black curve) and stimulated by cDBS for different widths of the interphase gap as indicated in the legend. The stimulation starts at $t=20\,{\rm{s}}$ as indicated by the vertical dashed line with parameter of the stimulation intensity $K=2$. (**B**–**E**) Examples of raster spike plots of STN neurons (blue dots) for (**B**) $K=0$ (no stimulation) and (**C**–**E**) $K=2$ and interphase gap (**C**) $GW=0\,{\rm{ms}}$, and (**D**) 2 ms, and (**E**) 5 ms. Black and red curves depict raw and filtered LFP, respectively, scaled by the factor 1000.

In this study we focus on the control of the collective synchronized dynamics of the STN-GPe network (1)–(4). The extent of synchronization can be estimated by the order parameter^50–52^

5 $$R(t)=|{N}^{-1}\,\sum _{j=1}^{N}\,\exp (i{\psi }_{j}(t))|,$$

where ${\psi }_{j}(t)$ are the phases of individual neurons calculated from the neuronal bursting dynamics. The phase ${\psi }_{j}(t)$ of the *j*th neuron attains the values ${\psi }_{j}({t}_{n})=2\pi n$, $n=0,1,\ldots$ at the time moments *t*_*n*_ of the burst onsets, i.e., the first spikes in the bursts, and linearly increases between two consecutive bursts ${\psi }_{j}(t)=2\pi (t-{t}_{n})/({t}_{n+1}-{t}_{n})+2\pi n$ for $t\in ({t}_{n},{t}_{n+1})$, $n=0,1,\ldots$^53^. The values of the order parameter *R*(*t*) range from 0 to 1 and correspond to the extent of in-phase synchronization in the population. Dynamics of the order parameter for the considered parameters of the stimulation-free synchronized STN neurons (${I}_{{\rm{stim}}}=0$ in equation (1)) is illustrated in Fig. 1A (black curve). The order parameter fluctuates around $R\approx 0.8$, which indicates a relatively strong in-phase synchronization of STN neurons.

The extent of synchronization is also reflected by the amplitude of the local field potential (LFP) which we model as an ensemble-averaged synaptic activity of neurons $LFP(t)={N}^{-1}\,{\sum }_{j=1}^{N}\,{s}_{j}$^54^, where ${s}_{j}(t)$ are the synaptic variables (4) of STN neurons, see also papers^55,56^ for a more sophisticated approach. The measured raw *LFP*(*t*) is on-line filtered by applying a linear damped oscillator

6 $$\ddot{u}+{\alpha }_{d}\dot{u}+{\omega }^{2}u={k}_{{\rm{f}}}LFP(t).$$

Parameter $\omega$ approximates the frequency of the LFP oscillations $\omega =2\pi /T$, where *T* is the mean period of the LFP. As the output signal of equation (6), that is the filtered LFP, we use the variable $x(t)=\dot{u}$, which has a zero phase shift with respect to the original LFP signal^35^. The damping and scaling coefficients in equation (6) were chosen as ${\alpha }_{d}={k}_{{\rm{f}}}=0.008$ which approximately preserves the amplitude of the input raw LFP signal^39^. Dynamics of raw and filtered LFP of STN neurons without stimulation is illustrated in Fig. 1B (black and red curves). The neurons exhibit in-phase synchronization and burst nearly simultaneously [Fig. 1B, blue dots], which is accompanied by large-amplitude LFP oscillations.

For the considered parameters, STN bursting neurons synchronize at ≈10 Hz (the mean period of LFP oscillations $T\approx 103\,{\rm{ms}}$) [Fig. 1B]. This frequency belongs to the frequency range 8–30 Hz which is referred to as basal ganglia beta frequency band, where an abnormal neuronal dynamics can be related to disease symptoms^22,57,58^. In particular, in parkinsonian monkeys the beta band extends to lower frequencies compared to in Parkinson’s patients^21^. The synchronization frequency is close to the low beta oscillatory range of 11–14 Hz, where the degree of synchronization suppression correlates with clinical motor improvement^59^. However, the considered model can also be used for computational investigation of other frequency bands, see Supplementary Fig. S2.

### HF DBS

**Figure 2:**
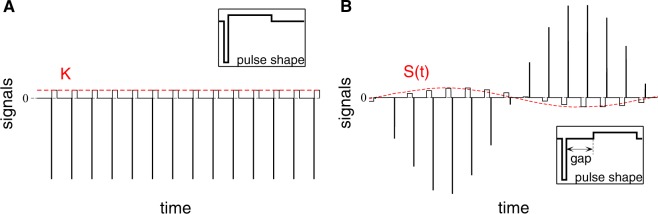
Stimulation signals of HF DBS and pulsatile delayed feedback. The amplitude of the high-frequency pulse train of charge-balanced asymmetric biphasic pulses (solid lines) is (**A**) kept constant and equal to the parameter of the stimulation intensity *K* for HF DBS stimulation or (**B**) modulated by a slowly oscillating smooth feedback signal *S*(*t*) (8) for pulsatile LDF stimulation depicted by red dashed curves. The shapes of single pulses are schematically illustrated in the inserts, which can contain an interphase gap between the cathodic and anodic phases of the pulse.

During HF DBS, STN neurons are stimulated by a train of high-frequency electrical biphasic charge-balanced pulses^2,60,61^. Each pulse consists of cathodic and anodic phases which deliver the same charge of opposite polarity providing, in such a way, a charge-balanced stimulation. This results in zero net charge injection into the stimulated tissue after each short biphasic pulse and prevents from injury to nervous tissue^2,42,43,62^. We consider asymmetric biphasic charge-balanced stimulation pulses used for the standard HF DBS^60,61^, which consist of a first short cathodic pulse (1st phase) followed by a longer charge-balancing 2nd phase of opposite polarity, see insert in Fig. 2A. We use the standard frequency of 130 Hz for the HF DBS pulse train (the inter-pulse interval $1000/130\approx 7.69\,{\rm{ms}}$)^60^ and the width of the short pulse (1st phase) *PW* = 0.2 ms that relates to the duration of its long counterpart as $1:10$ [Fig. 2A], which is found to be energy efficient^63^.

The stimulation current *I*_stim_ (in pA/*μ*m^2^) in equation (1) consists of a HF train of the above pulses of unit amplitude of the recharging 2nd phase amplified by a factor *A*(*t*)

7 $${I}_{{\rm{stim}}}(t)=A({t}_{n})\cdot \{\begin{array}{ll}-\,10, & {\rm{if}}\,{t}_{n}\le t < {t}_{n}+PW,\\ 0, & {\rm{if}}\,{t}_{n}+PW\le t < {t}_{n}+PW+GW,\\ 1, & {\rm{if}}\,{t}_{n}+PW+GW\le t < {t}_{n}+11PW+GW,\\ 0, & {\rm{otherwise}},\end{array}$$

for $t\in [{t}_{n},{t}_{n+1})$, where ${t}_{n}=1000n/F$ ms, $n=0,1,2,\ldots$ are the times of the pulse onsets [Fig. 2A], and *F* = 130 Hz is the frequency of the stimulation pulse train (number of pulses per second) as mentioned above. For HF DBS the factor *A*(*t*) = *K* is a constant dimensionless parameter of the stimulation intensity. Each pulse can contain an interphase time gap of width *GW* between the cathodic and anodic phases of the biphasic pulses, see insert in Fig. 2B, see also refs ^40,61,64,65^. For the considered pulse frequency and pulse width, the width of the interphase gap *GW* for charge-balanced pulses can range up to 5.49 ms, otherwise the recharging second phase of the pulses becomes too short to balance the charge imposed by the first pulse phase. For $GW > 7.49\,{\rm{ms}}$ the pulses turn to monophasic.

### Pulsatile delayed feedback stimulation

Neuronal synchronization of the considered model (1)–(4) can also be controlled by linear delayed feedback (LDF). This stimulation techniques has been suggested and investigated in the papers^28,29,36,39,40^. The feedback stimulation signal *S*(*t*) is calculated as^28,29,36,39,40^

8 $$S(t)=K\cdot (x(t-\tau )-x(t)),$$

where the signal $x(t)=\dot{u}$ is from equation (6) and represents the filtered LFP. Parameter *K* is a dimensionless feedback gain and, as before, will be referred to as parameter of the stimulation intensity, and $\tau$ is the stimulation delay.

Electrical stimulation of the brain with such a smooth signal might cause an irreversible charge deposit in the vicinity of the electrode and lead to a damage of the neuronal tissue^2,42,43^. This problem can be resolved as suggested in the recent papers^39,40^. We use the above high-frequency pulse train of biphasic charge-balanced pulses utilized for the standard HF DBS, whose amplitude is modulated by the slowly oscillating feedback signals *S*(*t*) as schematically illustrated in Fig. 2B, where an example of the pulsatile stimulation current *I*_*stim*_ in equation (1) of pulsatile LDF is shown. In equation (7) the amplification factor $A(t)=S(t)$ in this case. We refer to the stimulation with such pulse trains modulated by the smooth LDF signal *S*(*t*) as pulsatile LDF stimulation^39,40^.

### Demand-controlled adaptive stimulation

Together with continuous stimulation, where the stimulation signal is continuously delivered to the stimulated neurons, we also model a demand-controlled, adaptive stimulation, where the neurons are stimulated to the extent and when necessary in an intermittent way. Such a closed-loop stimulation can be adapted, for example, to the amount of the ongoing abnormal neuronal activity^7,13,15^, e.g., to the extent of synchronization of the stimulated neuronal population. Following clinical approaches^7,15^, the stimulation can intermittently be switched on and off, where the onsets and offsets of the stimulation can, e.g., be triggered by a threshold crossing by the local field potential (LFP) measured via the implanted electrode. We thus apply such an approach to the considered model of the STN-GPe neuronal network. We introduce two threshold values *Th*_on_ (upper threshold or on-threshold) and *Th*_off_ (lower threshold or off-threshold) with $T{h}_{{\rm{on}}}\ge T{h}_{{\rm{off}}}$ for the amplitude of the LFP. The stimulation will be switched on when the local maxima of the oscillating filtered LFP exceed the upper threshold *Th*_on_, and the stimulation will be switched off by setting the parameter of the stimulation intensity to $K=0$, when the LFP local maxima fall below the lower threshold *Th*_off_. We simulate and systematically compare continuous and adaptive HF DBS (cDBS and aDBS) as well as continuous and adaptive pulsatile LDF (cpLDF and apLDF) when the stimulation parameters such as stimulation intensity *K*, delay $\tau$, the width of the interphase gap *GW* and the LFP thresholds vary. For each condition and parameters we average the values of the calculated quantities (order parameter, stimulation time, amount of the administered stimulation etc., see below) over time after skipping a long enough transient as well as over several different simulations running with slightly different stimulation parameters. For the latter averaging we consider a few (10–20) slightly different time intervals ${T}_{{\rm{ramp}}}\in [0,2000]\,{\rm{ms}}$, where, at the stimulation onset, the parameter of the stimulation intensity *K* linearly increases from 0 to the corresponding indicated value, i.e., the stimulation intensity is linearly ramped up over slightly different ramping time intervals.

## Results

We compare the desynchronizing impact of the continuous and adaptive HF DBS to each other and to that of pulsatile LDF. The continuous smooth and pulsatile LDF administered to synchronized STN neurons has been investigated in refs ^39–41^ together with smooth and pulsatile nonlinear delayed feedback (NDF). In this paper the main attention is paid to suppression of synchronization by cDBS, aDBS, and apLDF.

### Adaptive HF DBS

The continuous and adaptive HF DBS is administered to synchronized STN population, where the neurons burst nearly simultaneously and exhibit a well-pronounced in-phase synchronization, see the raster spike plot in Fig. 1B. The order parameter fluctuates around a large value $\langle R\rangle \approx 0.8$ [Fig. 1A, black curve], and the LFP demonstrates large-amplitude oscillations [Fig. 1B, black and red curves]. Stimulation of the synchronized STN neurons by cDBS with the permanently delivered HF pulse train [Fig. 2A] of large enough stimulation intensity *K* can suppress the synchronization of STN neurons. During the stimulation, the order parameter *R* exhibits small values [Fig. 1A, red, green and blue curves], and the in-phase firing of the STN neurons is destroyed [Fig. 1C–E, blue dots], which is accompanied by a reduction of the LFP amplitude [Fig. 1C–E, black and red curves].

**Figure 3:**
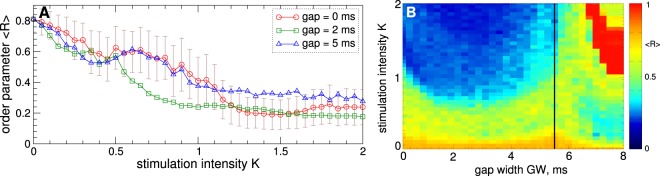
Impact of stimulation parameters on suppression of synchronization in the STN-GPe neuronal populations (1)–(4) by continuous HF DBS. (**A**) Averaged order parameter $\langle R\rangle$ versus stimulation intensity *K* for different interphase gaps as indicated in the legend. For zero gap the standard deviation of the order parameter fluctuations (plot (**A**), red curve) is indicated by error bars. (**B**) $\langle R\rangle$ depicted in color versus *K* and gap width *GW*. The vertical black line indicates the maximal value of $GW\approx 5.49\,{\rm{ms}}$ for charge-balanced stimulation pulses.

The desynchronizing impact of HF DBS depends on the stimulation parameters as illustrated in Fig. 3. Stronger stimulation can lead to a stronger desynchronization [Fig. 3A], and introducing an interphase gap may improve the desynchronizing impact of cDBS. For example, cDBS with gap width $GW=2\,{\rm{ms}}$ [Fig. 3A, green squares] can induce stronger desynchronization for a range of parameter *K* as compared to cDBS without gap [Fig. 3A, red circles]. Too large an interphase gap may not necessarily lead to an enhancement of desynchronization, see Fig. 3A for $GW=5\,{\rm{ms}}$ (blue triangles). As follows from the two-parameter diagram in Fig. 3B, there exists an optimal interphase gap, where, for fixed stimulation intensity *K*, cDBS can induce strongest desynchronization. On the other hand, synchronization can be suppressed for smaller *K* as it increases for an optimal gap. The considered gap $GW=2\,{\rm{ms}}$ is close to such an optimal value. Interestingly, the desynchronization region [Fig. 3B, blue domain] has a similar shape as the entrainment region of a single model neuron by pulses with gap in the presence of noise^65^. In our model such an entrainment manifests itself for large gap, where the pulses approach a monophasic shape, and stimulated neurons get synchronized by the stimulation [Fig. 3B, red domain].

**Figure 4:**
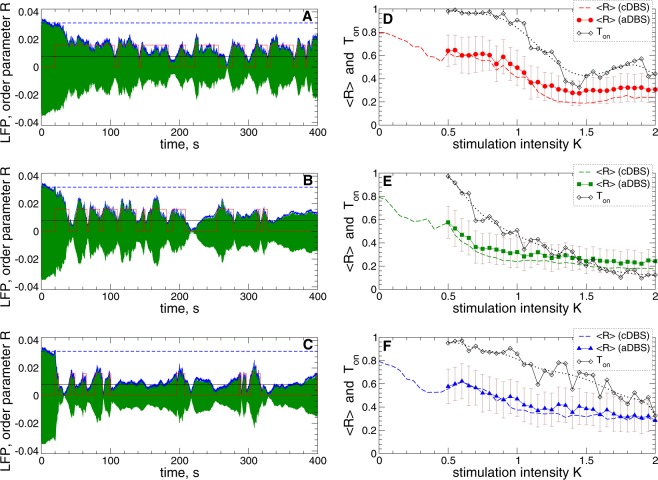
Suppression of synchronization in the STN-GPe neuronal populations (1)–(4) by adaptive HF DBS. (**A**–**C**) Time courses of the filtered LFP (green curves) and the order parameter $\langle R\rangle$ (blue curves, scaled by the factor 1/25) of STN neurons stimulated by aDBS started at $t=20\,{\rm{s}}$ for the interphase gap (**A**) $GW=0\,{\rm{ms}}$ and (**B**,**C**) $GW=2\,{\rm{ms}}$ and stimulation intensity (**A**,**B**) $K=1.2$ and (**C**) $K=2$. The scaled value of $\langle R\rangle$ of the synchronized and stimulation-free STN neurons [Fig. 1A, black curve, and Fig. 3A for $K=0$] is indicated by horizontal blue dashed lines for comparison. The red stepwise curves indicate the on- and off-epochs of aDBS for the LFP thresholds $T{h}_{{\rm{on}}}=0.016$ (upper value of the red stepwise curves) and $T{h}_{{\rm{off}}}=0.008$ (black horizontal line). (**D**–**F**) Averaged order parameter $\langle R\rangle$ versus stimulation intensity *K* for aDBS (filled symbols) and cDBS (dashed curves, copied from Fig. 3A for comparison) as indicated in the legends for the interphase gap (**D**) $GW=0\,{\rm{ms}}$, (**E**) 2 ms, and (**F**) 5 ms. The standard deviation of the order parameter fluctuations for aDBS is indicated by error bars. The fraction of the stimulation time *T*_on_ (black diamonds) of aDBS, where HF DBS was switched on, and its smoothed values (black dotted curves) are also shown.

The impact of aDBS on the collective dynamics of STN neurons is illustrated in Fig. 4. As explained in the Methods, during the adaptive stimulation the LFP is measured and filtered, and the stimulation is switched on and off if the values of the local maxima of the filtered LFP [Fig. 4A–C, green curves] exceed the upper threshold *Th*_on_ or fall below the lower threshold *Th*_off_ as illustrated by the red stepwise curves in Fig. 4A–C. The order parameter *R* [Fig. 4A–C, blue curves] closely follows the time course of the LFP amplitude if the former is scaled appropriately (by the factor 1/25 in this case) and demonstrates much smaller values as compared to the stimulation-free case [Fig. 4A–C, horizontal blue dashed lines]. We thus observe that such a stimulation strategy can result in synchronization suppression in the stimulated neuronal population in spite of an intermittent administration of the stimulation, where the stimulation time is significantly reduced.

As for the case of cDBS [Figs 1 and 3], introducing an interphase gap of an intermediate width can enhance the desynchronizing effect of aDBS, compare Fig. 4A for $GW=0\,{\rm{ms}}$ to Fig. 4B for $GW=2\,{\rm{ms}}$. Interestingly, such an improvement of the stimulation outcome is obtained at a substantial reduction of the stimulation time *T*_on_ that is the fraction of time, where the stimulation was switched on during aDBS. For example, for $K=1.2$, the averaged order parameter $\langle R\rangle \approx 0.33$ at ${T}_{{\rm{on}}}\approx 0.63$ for $GW=0$ ms in Fig. 4A and $\langle R\rangle \approx 0.29$ at ${T}_{{\rm{on}}}\approx 0.38$ for $GW=2\,{\rm{ms}}$ in Fig. 4B. For stronger stimulation, for example, for $K=2$ and $GW=2\,{\rm{ms}}$, the order parameter can reach smaller values $\langle R\rangle \approx 0.24$ obtained at even smaller stimulation time ${T}_{{\rm{o}}n}\approx 0.14$ [Fig. 4C].

We compare the desynchronizing impact of aDBS and cDBS by varying the stimulation intensity $K$ and plot the averaged order parameter $\langle R\rangle$ of the STN neurons stimulated by aDBS and cDBS in Fig. 4D–F versus parameter $K$. For the considered LFP thresholds $T{h}_{{\rm{on}}}=0.016$ and $T{h}_{{\rm{off}}}=0.008$, we found that the extent of the aDBS-induced desynchronization can approach that one induced by cDBS [Fig. 4D–F]. The desynchronization induced by aDBS can however be achieved at a much smaller amount of the stimulation time *T*_on_ as compared to cDBS [Fig. 4D–F, black diamonds], and a moderate interphase gap can strongly reduce *T*_on_. Too large an interphase gap may however not necessarily lead to an enhancement of the aDBS-induced desynchronization and a further decrease of the stimulation time [Fig. 4F]. For smaller LFP thresholds, e.g., for identical $T{h}_{{\rm{on}}}=T{h}_{{\rm{off}}}=0.01$, the aDBS-induced desynchronization can further be improved, and the order parameter nearly coincides with that induced by cDBS, especially, for the case of the interphase gap of intermediate width, see Supplementary Fig. S3. For such an optimal interphase gap also the amount of the stimulation time *T*_on_ is minimal as compared to other gaps [Fig. 4D–F and Supplementary Fig. S3].

**Figure 5:**
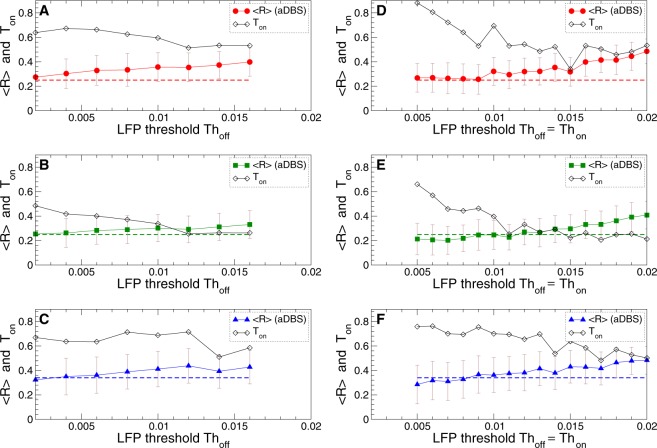
Impact of aDBS on the collective dynamics of STN-GPe neurons (1)–(4) for different LFP thresholds. The averaged order parameter $\langle R\rangle$ (filled red circles, green squares and blue triangles) and fraction of the stimulation time *T*_on_ (black diamonds) are plotted versus the lower LFP threshold *Th*_off_. The upper threshold is either fixed at (**A**–**C**) $T{h}_{{\rm{on}}}=0.016$ or (**D**–**F**) varied together with the lower threshold such that $T{h}_{{\rm{on}}}=T{h}_{{\rm{off}}}$. The standard deviation of the order parameter fluctuations is indicated by error bars. The horizontal dashed lines indicate the corresponding values of the order parameter $\langle R\rangle$ obtained by cDBS, see Fig. 3A for $K=1.2$. Interphase gap (**A**,**D**) $GW=0\,{\rm{ms}}$, (**B**,**E**) 2 ms, and (**C**,**F**) 5 ms. The stimulation intensity $K=1.2$.

The impact of aDBS on the synchronized dynamics of STN neurons depends on the LFP thresholds $T{h}_{{\rm{on}}}$ and $T{h}_{{\rm{off}}}$ as illustrated in Fig. 5. As a general tendency, when the LFP thresholds increase, the averaged order parameter $\langle R\rangle$ and, thus, the amount of the residual synchronization also increases. On the other hand, the fraction of the stimulation time ${T}_{{\rm{on}}}$ decreases at the same time. The neurons stimulated by aDBS are thus less desynchronized for large thresholds, but there exists a parameter range of small LFP thresholds, where aDBS suppresses the neuronal synchronization to the extent of cDBS or even slightly better [Fig. 5, cf. $\langle R\rangle$ for aDBS and cDBS]. For such parameters, however, aDBS is much more efficient in inducing desynchronization because a given extent of desynchronization can be obtained at a much smaller amount of the stimulation time (*T*_on_ is smaller than 1) and, thus, for much smaller amount of the administered stimulation current.

### Adaptive pulsatile linear delayed feedback

**Figure 6:**
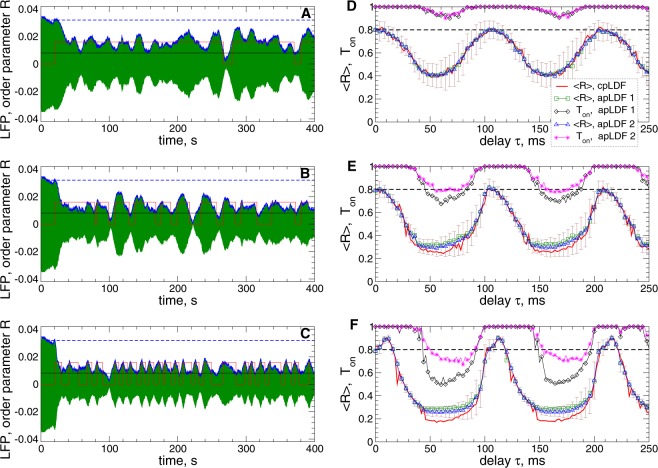
Suppression of synchronization in the STN-GPe neuronal populations (1)–(4) by apLDF. (**A**–**C**) Time courses of the filtered LFP (green curves) and the order parameter $\langle R\rangle$ (blue curves, scaled by the factor 1/25) of STN neurons stimulated by apLDF started at $t=20\,{\rm{s}}$ for the widths of the interphase gaps (**A**) $GW=0\,{\rm{ms}}$, (**B**) 2 ms, and (**C**) 5 ms. The scaled value of $\langle R\rangle$ of the synchronized and stimulation-free STN neurons [Figs 1A, black curve, and 3A for $K=0$] is indicated by horizontal blue dashed lines for comparison. The red stepwise curves indicate the on- and off-epochs of apLDF for the LFP thresholds $T{h}_{{\rm{on}}}=0.016$ (upper value of the red stepwise curves) and $T{h}_{{\rm{off}}}=0.008$ (black horizontal line). Parameters of the stimulation intensity $K=10$ and delay $\tau =60\,{\rm{ms}}$. (**D**–**F**) Averaged order parameter $\langle R\rangle$ versus stimulation delay $\tau$ for apLDF and cpLDF as indicated in the legend for the interphase gap (**D**) $GW=0\,{\rm{ms}}$, (**E**) 2 ms, and (**F**) 5 ms. The fraction of the stimulation time *T*_on_ of apLDF, where the stimulation was switched on is also shown. The horizontal dashed lines indicate the order parameter $\langle R\rangle$ of the stimulation-free STN neurons. Stimulation intensity $K=10$, and the LFP thresholds $T{h}_{{\rm{on}}}=0.016$ and $T{h}_{{\rm{off}}}=0.008$ for the case 1 (indicated as “apLDF 1” in the legend) and $T{h}_{{\rm{on}}}=T{h}_{{\rm{off}}}=0.01$ for the case 2 (indicated as “apLDF 2” in the legend). The standard deviation of the order parameter fluctuations in the latter case is indicated by error bars.

Examples of desynchronization by apLDF are illustrated in Fig. 6A–C for fixed stimulation intensity $K=10$ and delay $\tau =60\,{\rm{ms}}$ and for three widths of the interphase gap. For the considered parameters, the apLDF stimulation with the pulses containing an interphase gap [Fig. 2B] can have an enhanced desynchronizing impact on the stimulated neurons as compared to the case of zero gap. The order parameter $\langle R\rangle$ (and amplitude of the LFP) and the stimulation time ${T}_{{\rm{on}}}$ are better suppressed for larger gap. For example, $\langle R\rangle \approx 0.41$ at ${T}_{{\rm{on}}}\approx 0.95$ for $GW=0$ ms [Fig. 6A], $\langle R\rangle \approx 0.32$ at ${T}_{{\rm{on}}}\approx 0.67$ for $GW=2\,{\rm{ms}}$ [Fig. 6B], and $\langle R\rangle \approx 0.29$ at ${T}_{{\rm{on}}}\approx 0.5$ for $GW=5\,{\rm{ms}}$ [Fig. 6C].

The averaged order parameter $\langle R\rangle$ of the STN neurons stimulated by apLDF and cpLDF is depicted versus stimulation delay $\tau$ for fixed intensity $K=10$ in Fig. 6D–F for comparison. Two sets of the LFP thresholds for apLDF are considered, $T{h}_{{\rm{on}}}=0.016$ and $T{h}_{{\rm{off}}}=0.008$, and $T{h}_{{\rm{on}}}=T{h}_{{\rm{off}}}=0.01$, indicated as “apLDF 1” and “apLDF 2” in the legend, respectively. In the desynchronization regions, where the order parameter exhibits smaller values as compared to the stimulation-free case [Fig. 6D–F, black dashed lines], both cpLDF [Fig. 6D–F, red solid curves] and apLDF [Fig. 6D–F, green squares and blue triangles] stimulations induce stronger desynchronization as the width of the interphase gap increases. Moreover, a larger gap also leads to a substantial decrease of the stimulation time ${T}_{{\rm{on}}}$ for apLDF [Fig. 6D–F, black diamonds and magenta asterisks], as already mentioned above. Therefore, introducing an interphase gap in the stimulation pulses can lead to a stronger desynchronization by apLDF and to a simultaneous reduction of the stimulation time, and this effect gets more pronounced for larger gap. For the considered parameters, the extent of desynchronization induced by apLDF and cpLDF stimulations can be very close to each other [Fig. 6D,E]. However, the difference between them can get more pronounced if cpLDF-induced desynchronization is strong as, for example, for $GW=5\,{\rm{ms}}$ in Fig. 6F. Smaller LFP thresholds can further enhance the apLDF-induced desynchronization, but this can be achieved for a longer stimulation (larger ${T}_{{\rm{on}}}$) as illustrated in Fig. 6E,F, compare the cases “apLDF 1” and “apLDF 2”.

**Figure 7:**
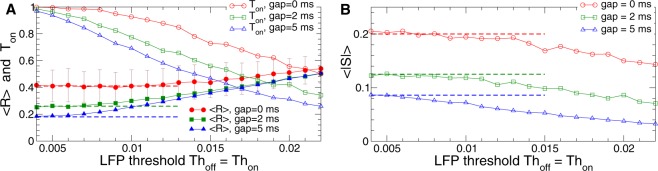
Desynchronization of STN-GPe neurons (1)–(4) by apLDF for different LFP thresholds. (**A**) The averaged order parameter $\langle R\rangle$ (filled symbols) of the STN neurons stimulated by apLDF and fraction of the stimulation time *T*_on_ (empty symbols) are depicted versus the LFP thresholds $T{h}_{{\rm{off}}}=T{h}_{{\rm{on}}}$ for different widths of the interphase gap as indicated in the legends. For zero gap the standard deviation of the order parameter fluctuations is indicated by error bars. The horizontal dashed lines indicate the corresponding values of the order parameter $\langle R\rangle$ induced by cpLDF, see Fig. 6D–F (red solid curves) for $\tau =60\,{\rm{ms}}$. (**B**) Amount of stimulation $\langle |S|\rangle$ administered by apLDF from plot (**A**), where the dashed lines indicate $\langle |S|\rangle$ for cpLDF. Stimulation intensity $K=10$ and delay $\tau =60\,{\rm{ms}}$.

As follows from Fig. 6D–F, apLDF has the same structure of the $(\tau ,K)$-parameter space as cpLDF reported in refs ^39,40^, where the desynchronization regions of small values of $\langle R\rangle$ periodically appear in the parameter space as $\tau$ increases with approximately mean LFP period $T\approx 103\,{\rm{ms}}$ of the synchronized stimulation-free neuronal ensemble as also found for other models^28,29,36^. For further analysis we fix a representative value of an optimal delay $\tau =60\,{\rm{ms}}$ (close to *T*/2) for strong desynchronization [Fig. 6D–F]. For fixed stimulation intensity $K=10$ and $\tau =60\,{\rm{ms}}$, the impact of the LFP thresholds on the desynchronizing outcome of apLDF is illustrated in Fig. 7A in more detail. We found that there is an apparent interdependency between the LFP thresholds, the extent of the stimulation-induced desynchronization as reflected by values of the order parameter $\langle R\rangle$ [Fig. 7A, filled symbols], and stimulation time ${T}_{{\rm{on}}}$ [Fig. 7A, empty symbols]. For larger LFP thresholds the stimulation with apLDF induces a weaker desynchronization ($\langle R\rangle$ increases), but this requires less stimulation time (${T}_{{\rm{on}}}$ decreases). On the other hand, if the LFP thresholds are small, apLDF better suppresses the neuronal synchronization which approaches the extent of desynchronization induced by cpLDF [Fig. 7A, dashed lines]. At this, the stimulation time increases toward ${T}_{{\rm{on}}}=1$ that corresponds to the case of continuous stimulation. Depending on the clinical needs and conditions, one could select the corresponding LFP thresholds to obtain the desirable extent of the stimulation-induced desynchronization as well as the amount of the stimulation time.

The discussed effects of the LFP thresholds are similar to both apLDF and aDBS techniques, see Figs 5 and 7A. For apLDF, larger interphase gap consistently leads to an enhancement the stimulation outcome [Fig. 7A], which may however not be the case for aDBS for too large an interphase gap [Fig. 5]. Another difference refers to the amount of the stimulation current administered to the stimulated neurons. In the case of aDBS it is directly proportional to the amount of the stimulation time for fixed stimulation intensity and will decay together with the stimulation time as LFP thresholds increase [Fig. 5]. For apLDF the situation is more complex since the amount of the administered stimulation calculated as time-averaged absolute value $\langle |S|\rangle$ of the feedback signal (8) used for modulation of the pulse amplitude [Fig. 2B] is proportional to both the LFP amplitude (extent of the stimulation-induced desynchronization) and the amount of the stimulation time ${T}_{{\rm{on}}}$. As LFP thresholds increase, the LFP amplitude increases together with the order parameter [Fig. 7A, filled symbols], whereas the amount of the stimulation time decreases [Fig. 7A, empty symbols]. Nevertheless, we found that for apLDF the amount of the administered stimulation $\langle |S|\rangle$ monotonically decays together with the stimulation time as the LFP thresholds increase, see Fig. 7B.

**Figure 8:**
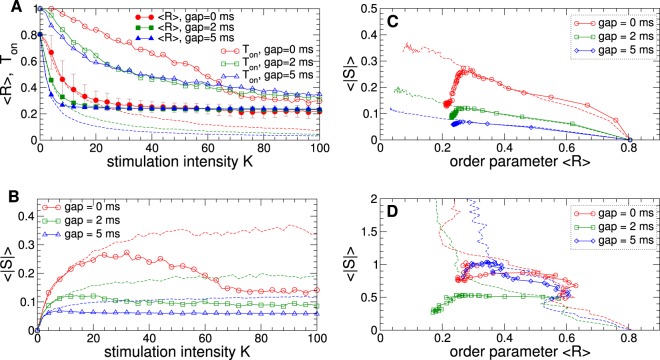
Stimulation outcome of the apLDF stimulation and aDBS administered to STN-GPe neurons (1)–(4). (**A**,**B**) The averaged order parameter $\langle R\rangle$ of the STN neurons stimulated by apLDF, fraction of the stimulation time *T*_on_ and amount of stimulation $\langle |S|\rangle$ administered by apLDF versus parameter *K* of the stimulation intensity for different widths of the interphase gap as indicated in the legends. Dashed thin curves of the same color depict the corresponding values of (**A**) the order parameter $\langle R\rangle$ and (**B**) administered amount of stimulation $\langle |S|\rangle$ of cpLDF. For zero gap the standard deviation of the order parameter fluctuations is indicated by error bars. (**C**,**D**) Administered amount of stimulation $\langle |S|\rangle$ versus the reached extent of the stimulation-induced desynchronization as given by values of $\langle R\rangle$ for (**C**) apLDF stimulation and (**D**) aDBS for the interphase gaps indicated in the legends. Results for conventional cpLDF and cDBS are depicted by dashed curves of the corresponding color. Stimulation delay $\tau =60\,{\rm{ms}}$, and LFP thresholds $T{h}_{{\rm{on}}}=T{h}_{{\rm{off}}}=0.01$.

Increasing stimulation intensity *K* can lead to a more pronounced desynchronization induced by apLDF and cpLDF as illustrated in Fig. 8A, where the order parameter decays as parameter *K* increases. For apLDF and fixed LFP thresholds, the order parameter saturates at some value for large *K* [Fig. 8A, filled symbols]. The stimulation time ${T}_{{\rm{on}}}$, however, further decreases for large stimulation intensity. Based on such a behavior of the order parameter and simulation time, the amount of the stimulation administered by apLDF remains bounded in spite of increasing stimulation intensity and demonstrates a non-monotonic behavior as illustrated in Fig. 8B. Moreover, for the same large enough values of *K*, the apLDF stimulation delivers less stimulation current as compared to cpLDF stimulation, see Fig. 8B.

To evaluate and illustrate the efficiency of apLDF and cpLDF in inducing desynchronization, we plot in Fig. 8C the amount of the administered stimulation $\langle |S|\rangle$ versus the extent of the reached stimulation-induced desynchronization as given by the values of the averaged order parameter $\langle R\rangle$. For cpLDF [Fig. 8C, dashed curves] stronger desynchronization (smaller $\langle R\rangle$) can be obtained for larger amount of the stimulation, whereas the latter can significantly be reduced by introducing and increasing an interphase gap in the stimulation pulses. Utilizing the discussed on-off strategy for apLDF can further diminish the amount of the stimulation necessary to obtain a given level of desynchronization such that $\langle |S|\rangle$ starts to decay together with the order parameter [Fig. 8C, solid curves with symbols]. The order parameter $\langle R\rangle$ can however be bounded to some moderate values since is saturates with increasing stimulation intensity *K* for fixed LFP thresholds [Fig. 8A]. Therefore, apLDF can be very efficient in inducing a moderate desynchronization, while the LFP thresholds have to be reduced or the stimulation can be switched to a conventional cpLDF when a much stronger desynchronization is required.

The efficiency of aDBS and conventional cDBS in suppressing synchronization is illustrated in Fig. 8D, where, by analogy with LDF stimulation, the amount of the administered stimulation $\langle |S|\rangle$ is calculated from the signal $S(t)=K$ which is used to modulate/define the amplitude of the stimulation pulses [Fig. 2A] similar to the feedback stimulation with oscillating signal *S*(*t*) [Fig. 2B]. The conventional cDBS administers much more stimulation current to obtain a given extent of desynchronization as compared to cpLDF, compare dashed curves in Fig. 8C,D (notice the difference in scaling by vertical axes). Introducing an interphase gap of a moderate width may be beneficial for the stimulation efficiency of cDBS, compare dashed red curve for $GW=0\,{\rm{ms}}$ to the dashed green curve for $GW=2\,{\rm{ms}}$ in Fig. 8D. Too large a gap, however, may not necessarily lead to a more efficient desynchronization [Fig. 8D, dashed blue curve for $GW=5\,{\rm{ms}}$]. The on-off aDBS can induce at least the same extent of desynchronization as cDBS, but for much smaller amount of the administered stimulation [Fig. 8D, solid curves with symbols]. This is especially well pronounced for the interphase gap of moderate width, for example, for $GW=2\,{\rm{ms}}$ [Fig. 8D, green squares]. For such parameters the efficiency of aDBS may approach that of pulsatile LDF, albeit aDBS still remains less efficient than pulsatile LDF. For instance, to obtain desynchronization with $\langle R\rangle \approx 0.24$, cpLDF and apLDF require $\langle |S|\rangle \approx 0.08$ and 0.06 for $GW=5\,{\rm{ms}}$, respectively, whereas the smallest amount of the stimulation for cDBS and aDBS $\langle |S|\rangle \approx 1.0$ and 0.5 for $GW=2\,{\rm{ms}}$, respectively. For stronger desynchronization with $\langle R\rangle \approx 0.17$ obtained by aDBS for $GW=2\,{\rm{ms}}$ in Fig. 8D (green squares), the amount of the administered stimulation $\langle |S|\rangle \approx 0.27$ for aDBS (2.0 for cDBS), whereas $\langle |S|\rangle \approx 0.34$, 0.15, and 0.085 for cpLDF for *GW* = 0 ms, 2 ms, and 5 ms, respectively. aDBS can thus be a much more efficient stimulation technique for suppression of abnormal neuronal synchronization as compared to the conventional HF DBS. The stimulation efficiency can further be enhanced when pulsatile feedback techniques (cpLDF or apLDF) is used for desynchronization.

### Dynamics of the LFP amplitude

**Figure 9:**
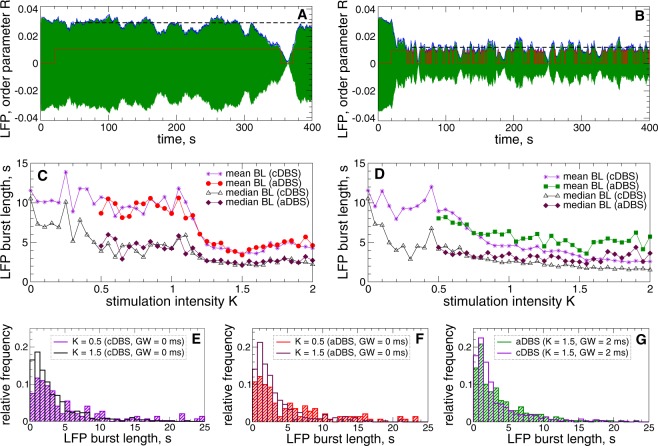
Modulation of the amplitude dynamics of STN LFP by HF DBS. (**A**,**B**) Time courses of the filtered LFP (green curves) and the smoothed order parameter *R* (blue curves) of STN neurons (1)–(4) stimulated by aDBS with the interphase gaps $GW=0\,{\rm{ms}}$ and stimulation intensity (**A**) $K=0.5$ and (**B**) $K=1.5$. The values of the order parameter are scaled by the factor 1/25. The stimulation starts at $t=20\,{\rm{s}}$. The red stepwise curve indicates the on- and off-epochs of aDBS. The LFP thresholds for aDBS $T{h}_{{\rm{on}}}=T{h}_{{\rm{off}}}=0.01$ (upper value of the red stepwise curves). The black dashed lines indicate the 65-percentile of the order parameter time variation starting from $t=50\,{\rm{s}}$. (**C**,**D**) Mean and median values of the LFP burst length versus parameter of the stimulation intensity *K* for cDBS and aDBS as indicated in the legend (BL stands for “burst length”) and for the interphase gap (**C**) $GW=0\,{\rm{ms}}$ and (**D**) 2 ms. (**E**–**G**) Examples of the LFP burst length distribution for cDBS and aDBS illustrated by frequency histograms for two values of *K* and *GW*, as indicated in the legends.

Along with synchronization suppression by cDBS and aDBS and reduction of the LFP amplitude [Figs 1, 3–5], the dynamics of the LFP undergoes additional modification, which is illustrated in Fig. 9. We found that LFP fluctuations start to exhibit many short bursts as the stimulation intensity increases, see Fig. 9A,B. To detect such bursts, we proceed as suggested in the recent paper^19^ and define a burst threshold being a 75-percentile of the LFP amplitude variation. Then the burst onset is detected at the time moment of the upward crossing of this threshold by the LFP amplitude. Since the values of the order parameter *R*(*t*) closely approximate the variation of the LFP amplitude [Figs 4A–C, 6A–C and 9A,B], we use the time courses of *R*(*t*) for such calculations. To ameliorate an overestimation of the number of short bursts that could be detected due to small noisy fluctuations of the order parameter, we smoothed the time courses of *R*(*t*) by using a moving average over 400 ms with the step of 10 ms and introduce a lower threshold of 65-percentile as well. Then the onsets and offsets of the LFP bursts were detected when the smoothed time signal of the order parameter [Fig. 9A,B, blue curves] crosses the upper and lower thresholds which are its 75- and 65-percentiles [Fig. 9A,B, black dashed lines], respectively.

The LFP dynamics induced by aDBS and cDBS apparently demonstrates a tendency toward a prevalence of short bursts when the stimulation gets stronger as illustrated in Fig. 9C,D. The mean and median of the burst length decay as parameter *K* increases and can show either a rather pronounced fast transition at a certain critical stimulation intensity as for zero interphase gap [Fig. 9C] or gradually decrease as for the gap width $GW=2\,{\rm{ms}}$ [Fig. 9D]. Based on our simulations of the considered model, the behavior and properties of the LFP bursts appear to be similar for both stimulation modalities, where cDBS can cause slightly shorter bursts than aDBS, especially, for large *K* and non-zero gap. The difference between the mean and median of the burst lengths is also similar for cDBS and aDBS, which indicates a relatively strong asymmetry in their distributions. Indeed, the distribution histograms are skewed toward long bursts as illustrated in Fig. 9E–G. For stronger stimulation the LFP bursts distribute more densely near short bursts such that the relative number of the bursts shorter than, for example, 5 s increases from 54% to 79% for cDBS [Fig. 9E] and from 52% to 81% for aDBS [Fig. 9F] when *K* grows from 0.5 to 1.5. For the case of non-zero gap $GW=2$, the situation is similar, where approximately 80% and 73% of the LFP bursts are shorter than 5 s for cDBS and aDBS, respectively, for $K=1.5$ [Fig. 9G]. We thus showed that aDBS and cDBS can significantly shorten the LFP bursts, which may have a therapeutic effect as discussed in ref. ^19^.

**Figure 10:**
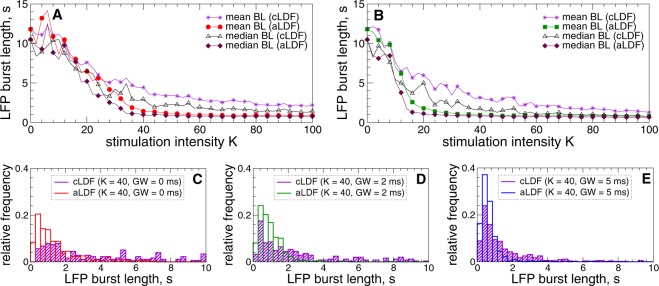
Modulation of the amplitude dynamics of STN LFP by pulsatile LDF administered to STN-GPe neurons (1)–(4). (**A**,**B**) Mean and median values of the STN LFP burst length versus stimulation intensity *K* for cpLDF and apLDF as indicated in the legend (BL stands for “burst length”) and for the interphase gap (**A**) $GW=0\,{\rm{ms}}$ and (**B**) 2 ms. (**C**,**D**) Examples of the LFP burst length distribution for cpLDF and apLDF illustrated by frequency histograms for $K=40$ and three values of *GW*, as indicated in the legends. LFP thresholds for apLDF $T{h}_{{\rm{on}}}=T{h}_{{\rm{off}}}=0.01$ and stimulation delay $\tau =60\,{\rm{ms}}$.

For pulsatile LDF stimulation the situation is similar, where stronger stimulation with larger intensity *K* also shortens the LFP bursts as illustrated in Fig. 10. The main difference to HF DBS is however that apLDF stimulation induces shorter LFP bursts than cpLDF [Fig. 10], which is statistically significant with $p < 0.001$ for $K > 32$ at $GW=0\,{\rm{ms}}$, $K > 14$ at $GW=2\,{\rm{ms}}$, and $K > 8$ at $GW=5\,{\rm{ms}}$, see Supplementary Fig. S5. Moreover, the distributions of the burst length become more symmetrically localized at short bursts as *K* increases, where the mean and median values approach each other [Fig. 10A,B], especially, for apLDF stimulation and large interphase gap [Fig. 10C–E].

## Discussion

In this computational study we investigated the desynchronizing effects of different continuous and adaptive stimulation techniques, cDBS, aDBS, cpLDF, and apLDF, on excessively synchronized populations of STN-GPe model neurons. Desynchronization might matter for the following reason. Prior to the first aDBS approaches^5–20^, a number of closed-loop demand-controlled desynchronizing DBS techniques were developed computationally^66–68^. Taking into account spike timing-dependent plasticity (STDP)^69,70^ it was computationally shown that desynchronizing coordinated reset (CR) stimulation may shift networks from attractors with strong synaptic connectivity and strong neural synchrony to attractors with weak synaptic connectivity and weak synchrony^71–73^. The same desynchronizing stimulation technique (CR-DBS) caused long-lasting therapeutic effects in parkinsonian monkeys^74,75^ as well as in Parkinson’s patients with CR-DBS^58^. In contrast, therapeutic effects of cDBS vanish after cessation of stimulation in both parkinsonian MPTP monkeys^74,75^ and PD patients^76,77^. Together with cDBS and aDBS we therefore considered pulsatile LDF stimulation^39–41^ that, unlike cDBS, was initially designed to counteract synchronization by desynchronization^28,29,36^.

We studied how the performance of the considered stimulation techniques depends on parameters and, in particular, showed that increasing stimulation intensity *K* leads to a stronger suppression of the abnormal neuronal synchronization by cDBS and aDBS [Figs 3 and 4]. At this a required clinical effect (RCE) can be achieved at some value of *K*, which is a lower boundary value of the stimulation intensity of the therapeutic window as known from clinical results^78^. For example, a 50% reduction of the beta-band LFP amplitude could be sufficient for a satisfactory clinical effect^7,15,19^. Introducing an interphase gap of moderate width of, e.g., 2 ms to the stimulation pulses can widen the therapeutic window by decreasing its lower boundary from $K\approx 1$ for zero gap to $K\approx 0.6$ for the gap width $GW=2\,{\rm{ms}}$ [Fig. 3A]. The same conclusion can be made for aDBS, where an interphase gap of moderate width and stronger stimulation could also lead to a shorter stimulation time [Fig. 4 and Supplementary Fig. S3]. The LFP amplitude thresholds used for triggering aDBS are also important parameters. At smaller thresholds aDBS tends to induce better desynchronization [Figs 4, 5 and Supplementary Fig. S3] such that aDBS can be at least as effective as cDBS or even better. In our model enhanced desynchronization for smaller thresholds, however, requires more stimulation time such that optimal values of parameters could be selected based on a trade-off between stronger desynchronization and shorter stimulation time. Again, an interphase gap of moderate width is favorable and enables a stronger desynchronization for smaller stimulation time compared to the case of zero gap [Figs 4, 5 and Supplementary Fig. S3].

The mechanism and the beneficial effect of the interphase gap can be explained based on the modeling and experimental results^43,65,79^ showing that it can reduce the counteracting impact of the recharging phase on the stimulation effect induced by the first phase of the pulses. Hence, this mechanism appears to be model independent and, as we showed, governs the enhancement of the stimulation-induced desynchronization when an interphase gap is introduced in the stimulation pulses. We observe a similar favorable effect when the duration of the recharging phase increases, as illustrated in Supplementary Fig. S4. This results in a larger difference in the amplitudes of the two phases of the stimulation pulses and reduces the counteracting effect of the second, recharging pulse phase as in the case of the interphase gap, in agreement with other studies^43,65,79^.

We computationally compared aDBS/cDBS with apLDF/cpLDF. For apLDF we observed the same threshold dependence as for aDBS: The extent of the apLDF-induced desynchronization approaches the cpLDF level for small LFP thresholds [Fig. 7]. At this, however, the relative stimulation time may quickly reach values close to 100%, which turns apLDF to cpLDF. We also estimated the amount of the stimulation current administered by adaptive stimulation, which depends on the stimulation time. For aDBS the amount of administered stimulation is simply proportional to the average stimulation time. For apLDF the situation is more complex, because the feedback signal (8) also depends on the LFP signal. Nevertheless, we found that the amount of the stimulation decays together with the stimulation time as the LFP thresholds increase [Fig. 7B], and apLDF may also administer less stimulation for large stimulation intensity *K* [Fig. 8B]. In contrast, cpLDF delivers more stimulation current as *K* increases, which also results in stronger desynchronization [Fig. 8], see also refs ^39,40^.

We also showed that apLDF is more efficient in suppressing abnormal neuronal synchronization than cpLDF, where apLDF delivers significantly less stimulation current for the same extent of the stimulation-induced desynchronization [Fig. 8C]. However, if a strong desynchronization is required, cpLDF can also be a good candidate. For such a situation pulsatile nonlinear delayed feedback may also be appropriate and cause an even more efficient desynchronization^40^. A great enhancement in efficiency can also be observed for aDBS as compared to cDBS [Fig. 8D], where the amount of the stimulation administered by aDBS can be several times smaller than for cDBS. With such an improvement, aDBS approaches the pulsatile LDF in its efficiency in suppressing abnormal neuronal dynamics, in particular, for the interphase gap of intermediate width, albeit the latter stimulation technique still remains to be more efficient.

With the considered model we tested the effects observed in electrophysiological data^19^ and also found that aDBS reduced the length of beta band bursts. We however revealed the same effect for cDBS [Fig. 9], which was not observed in patient data^19^ and might indicate that a more sophisticated model is necessary to account for such a difference, see discussion below. Nevertheless, in the considered model apLDF causes a significantly stronger reduction of LFP burst length than cpLDF [Fig. 10], i.e., adaptive stimulation more strongly reduces LFP burst length than continuous stimulation as suggested in ref. ^19^ for aDBS/cDBS. Future studies might also consider the impact on gamma power and gamma burst rate^80^.

In the computational model employed here^44,45^, cDBS and aDBS cause a desynchronization. However, there is no consensus on desynchronization being the mechanism of action of cDBS^81–84^. A large number of studies favor excitation or, conversely, depolarization blockade, inhibition, synaptic inhibition or synaptic depression, disruption (as opposed to desynchronization), jamming, and stimulation-induced modulation of pathological network activity or other processes as mechanisms of DBS^81–84^. By a similar token, several computational studies, performed in qualitatively different computational models, revealed a number of cDBS mechanisms that were qualitatively different from desynchronization^85–88^. Other modeling studies^86,89,90^ reported desynchronizing effects of cDBS as observed in this study. In particular, an interplay between inhibitory and excitatory effects of the stimulation may support the desynchronizing impact of cDBS^86^, which we also observed in the considered model, where both excitatory (STN) and inhibitory (GPe) neuronal populations participate in establishing such a stimulation-induced desynchronized regime. The mechanism of LDF with smooth stimulation signal is, on the other hand, relatively well understood, where the parameter regions of perfect desynchronization with vanishing mean field are bounded by bifurcation curves^28,29,91^. The desynchronization mechanism of LDF was also investigated for a pulsatile stimulation signal and for the considerably more complex model used in this study, where a similar shape of the desynchronization regions was revealed^39,41^.

The closed-loop (delayed-feedback) techniques were investigated in this study in the framework of a top-down approach, where they were first introduced and studied in simple models^28,29,36^, and then the obtained predictions were tested in more realistic models of increasing complexity and for more realistic stimulation setups^39–41^. Comparing the differences in the stimulation outcome of simple and more complex models could help to evaluate the important factors shaping the model response in the latter case. Such a model-based approach is aimed to assess and optimize the effects of DBS configurations, where both open-loop and closed-loop setups received much attention, see, for example, refs ^28–41,45,66–68,71–73,85–90^, and recent comprehensive reviews^92,93^ and references therein. Several sophisticated closed-loop control designs were suggested for DBS based on modulation of the stimulation waveform including stimulation timing, amplitude, spatiotemporal patterns of stimulation, and other parameters^94–100^. For this, the stimulation (feedback) goals can include the prescribed (healthy) activity patterns of the stimulated neuronal population, restoration of thalamocortical relay reliability, suppression of abnormal beta oscillations to mention a few. In this study we considered a simple on-off adaptive stimulation pattern already realized in clinical setups^5–12,14–16,18–20^ and aimed at inducing desynchronization, which automatically leads to suppression of pathological neuronal (beta-band) oscillations. Future studies may also be devoted to comparisons and combinations of different DBS optimization procedures, involving, for instance, variations of the temporal stimulation pattern with model-based computational evolution^101^.

The closed-loop stimulation approach presented here assumes that abnormal neuronal synchronization can be recorded reliably and represents disease-related abnormal processes and symptoms in the individual patient to a sufficient degree^102,103^. For a number of reasons, however, it is a matter of debate whether, for instance, beta band oscillations might serve as such a biomarker for feedback stimulation^14,102–108^, and, for example, cortical gamma oscillations can be incorporated in the feedback loop^109^, see also discussion in^40,41^.

For the next step in the top-down approach it is important to consider a realistic 3-Dim reconstruction of STN and GPe^73^ and accordingly increase the number of neurons^110^, which is expected to enhance the observed stimulation-induced desynchronization^28^. Since STN and GPe are influenced by the dynamics of larger circuits that involve the entire motor loop, other brain structures should also be incorporated in the models^45,97,111,112^. For example, the striatal input to GPe plays a pivotal role, in part because of the strengthening of the GPe synapses^49^. More realistic and physiologically motivated connectivity patterns, including intra-nuclear coupling, can also be considered^44,45,113–115^. The stimulation-induced desynchronization of STN-GPe activity can be beneficial if it also spreads to internal globus pallidus (GPi) and pallido-thalamic pathways and improves thalamic relay reliability^45^. Furthermore, antidromic activation of GPe projections and reentrant and reinforcement effects of HF DBS along the entire basal ganglia-thalamo-cortical motor loop may play an important role in the mechanism of DBS^83,111^. It is therefore necessary to extend the model from an isolated STN-GPe network to larger circuits^45,92,93,97,111,112^ and, for instance, take into account the cortical involvement in the abnormal synchronization process^59^. However, the more detailed and complex a model is, the more difficult it gets to perform a systematic analysis of its dynamics and obtain reasonably reliable and general predictions.

## Supplementary information

Supplementary Information
