# Supplementary material for: Adaptive delivery of continuous and delayed feedback deep brain stimulation - a computational study
Source: Sci Rep. 2019 Jul 22;9:10585. doi: 10.1038/s41598-019-47036-4 (PMC6646395; doi:10.1038/s41598-019-47036-4)
Supplement: Supplementary file 1 — Supplementary Information [file 41598_2019_47036_MOESM1_ESM.pdf]

Adaptive delivery of continuous and delayed feedback deep brain stimulation - a computational study

Oleksandr V. Popovych[1,*] **and Peter A. Tass**[2]

[1]Institute of Neuroscience and Medicine - Brain & Behaviour (INM-7), Research Centre Juelich, Juelich, Germany
[2]Department of Neurosurgery, Stanford University, Stanford, California, United States
[*]o.popovych@fz-juelich.de

Supplementary information

Parameter	STN	GPe	Units	Parameter	STN	GPe	Units
g_L	2.25	0.1	nS/μm^2	θ_m	-30.0	-37.0	mV
g_K	40	30	nS/μm^2	θ_h	-39.0	-58.0	mV
g_{Na}	50	120	nS/μm^2	θ_n	-32.0	-50.0	mV
g_T	0.5	0.5	nS/μm^2	θ_r	-67.0	-70.0	mV
g_{Ca}	0.5	0.15	nS/μm^2	θ_a	-63.0	-57.0	mV
g_{AHP}	9.0	30	nS/μm^2	θ_b	0.4	–	
v_L	-60.0	-55.0	mV	θ_s	-39.0	-35.0	mV
v_K	-80.0	-80.0	mV	θ_h^τ	-57.0	-40.0	mV
v_{Na}	55.0	55.0	mV	θ_n^τ	-80.0	-40.0	mV
v_{Ca}	140.0	120.0	mV	θ_r^τ	68.0	–	mV
τ_h^1	500.0	0.27	ms	θ_g^H	-39.0	-57.0	mV
τ_n^1	100.0	0.27	ms	θ_g	30.0	20.0	mV
τ_r^1	17.5	–	ms	σ_m	15.0	10.0	mV
τ_h^0	1.0	0.05	ms	σ_h	-3.1	-12.0	mV
τ_n^0	1.0	0.05	ms	σ_n	8.0	14.0	mV
τ_r^0	40.0	–	ms	σ_r	-2.0	-2.0	mV
ϕ_h	5	0.1		σ_a	7.8	2.0	mV
ϕ_n	5	0.3		σ_b	-0.1	–	
ϕ_r	2	1.0		σ_s	8.0	2.0	mV
k_1	15.0	30.0		σ_h^τ	-3.0	-12.0	mV
k_{Ca}	22.5	3.0		σ_n^τ	-26.0	-12.0	mV
σ_r^τ	-2.2	–	mV	σ_g^H	8.0	2.0	mV
α	5.0	2.0	ms^{-1}	β	1.0	0.045	ms^{-1}
$I_{app,j}$	$\mathcal{N}(10, 0.015^2)$	-4.24	pA/μm^2	τ_r	–	30	ms
C_m	1.0	1.0	pF/μm^2	ε_j	$5\cdot10^{-5}$	$\mathcal{N}(0.0055, [2\cdot10^{-5}]^2)$	ms^{-1}

Table S1. Parameter set of the considered model of STN-GPe network (1)-(4).

The values of parameters of the neuronal model (1) - (4) are listed in Table S1. The model was originally introduced in paper[1]. For STN neurons, the values of parameters g_K, g_{Na}, ϕ_h, ϕ_n, ϕ_r, $I_{app,j}$ (Gaussian distributed in our case), and ε_j are taken from the paper[2]. For GPe neurons, the values of parameters ϕ_h, ϕ_n, k_{Ca}, and ε_j (Gaussian distributed in our case) are again

taken from the paper[2], where parameters $I_{\mathrm{app},j}$ and β are slightly modified to demonstrate the synchronous bursting dynamics of the STN neurons, which is a hallmark of the pathological parkinsonian state[3,4]. The values of the other parameters are taken from the original paper[1]. As in the paper[1], we model the input to the GPe cells from the striatum by a constant hyperpolarizing current $I_{\mathrm{app},j}$ and assume that it is strong enough such that GPe neurons are not spontaneously active and generate rhythmic discharges due to STN-GPe coupling.

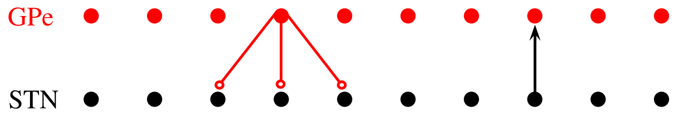

Figure S1. Coupling pattern of the considered STN-GPe neuronal network (1)-(4). Black circles depict excitatory STN neurons, and red circles depict inhibitory GPe neurons. Each STN neuron excites a single GPe cell, whereas each GPe cell inhibits three STN neurons. This simplified connectivity pattern was considered in the paper[2], where the synchronized dynamics generated by the model (1)-(4) was nevertheless well matched with experimental data from parkinsonian patients.

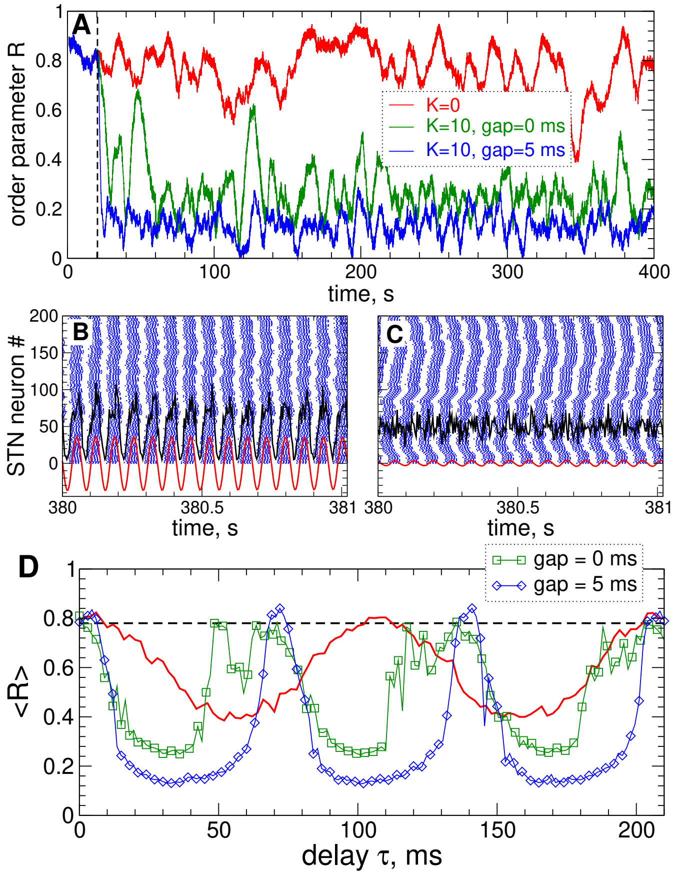

Figure S2. Suppression of synchronization in the STN-GPe neuronal populations (1) - (4) with scaled time $t' = t/1.5$ by continuous pLDF. (**A**) Time courses of the order parameter R of STN neurons stimulated by cpLDF are depicted for different widths of the interphase gap $GW = 0$ ms (green curve) and 5 ms (blue curve) as indicated in the legend. The stimulation starts at $t = 20$ s as indicated by the vertical dashed line with parameter of the stimulation intensity $K = 10$. The upper red curve depicts the order parameter of the stimulation-free ($K = 0$) STN neurons. (**B**), (**C**) Examples of raster spike plots of STN neurons (blue dots) for (**B**) $K = 0$ (no stimulation) and (**C**) $K = 10$ and interphase gap $GW = 5$ ms. Black and red curves depict raw and filtered LFP, respectively, scaled by the factor 1000. Stimulation delay $\tau = 33$ ms in plots (**A**) and (**C**). (**D**) Averaged order parameter $\langle R \rangle$ versus stimulation delay τ for fixed $K = 10$ and for different interphase gaps as indicated in the legend. Coupling parameter $g_{\mathrm{G}\to\mathrm{S}} = 1.34$ nS/μm^2. Red curve in plot (**D**) is copied from Figs. 6A and S4A for comparison.

In Fig. S2 we illustrate that the considered system (1) - (4) can be used to model the neuronal synchronization of STN-GPe neurons in other frequency bands. For the considered parameters, the model demonstrates an intrinsic synchronized dynamics at the frequency ≈ 10 Hz (mean LFP period $T \approx 103$ ms), see Fig. 1B, which belongs to the frequency range $8-35$ Hz, where an abnormal neuronal dynamics can be related to disease symptoms[3,5,6]. This frequency is close to the the low beta oscillatory range of $11-14$ Hz, where the degree of suppression of synchronization correlates with clinical motor improvement[7]. However, in PD patients the peaks of the STN LFP power can be observed at higher beta-band frequencies larger than 13 Hz[5,6,8]. The considered neuronal model can be modified to investigate and control of such high-frequency beta-band oscillations of the LFP via a top-down approach by a simple rescaling of time $t' = t/A$, where the LFP oscillations can be accelerated by the factor A. For example, for $A = 1.5$ the burst synchronization of STN neurons in the model takes place at the frequency ≈ 15 Hz with mean LFP period $T \approx 68$ ms [Fig. S2B]. The neuronal synchronization can be suppressed by pulsatile LDF stimulation [Fig. S2A,C], where introducing an interphase gap can improve the stimulation outcome [Fig. S2A,D] as for the case considered in the paper. The desynchronization parameter regions follow the same rule as reported in the paper and emerge centered around $\tau = T/2 + nT$, $n = 0, 1, 2, \ldots$ They thus appear more frequently in the parameter space as the stimulation delay τ increases since T is smaller. We thus expect that the reported results can also be obtained for neuronal synchronization at higher beta-band frequencies, and the derived conclusions remain valid also in this case.

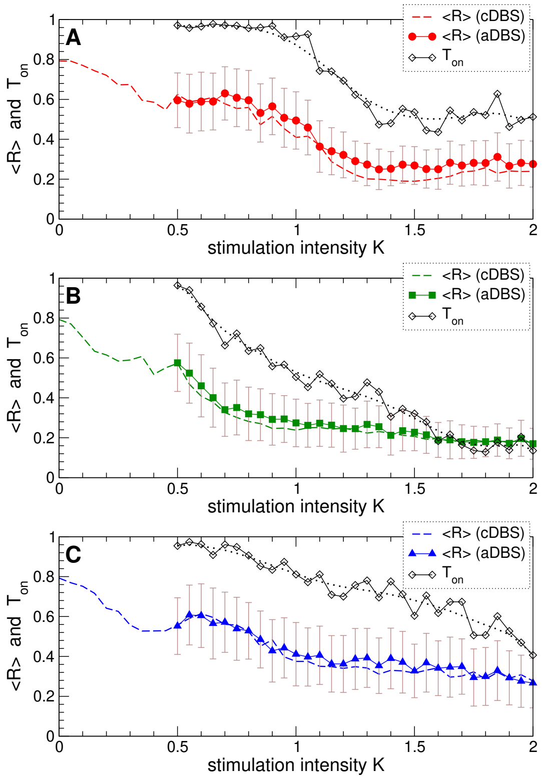

Figure S3. Suppression of synchronization in the STN-GPe neuronal populations (1)-(4) by adaptive HF DBS. Averaged order parameter $\langle R \rangle$ is depicted versus stimulation intensity K for aDBS (filled symbols) and cDBS (dashed curves) given for comparison as indicated in the legends for the interphase gap (**A**) $GW = 0$ ms, (**B**) 2 ms, and (**C**) 5 ms. The standard deviation of the order parameter fluctuations for aDBS is indicated by error bars. The fraction of the stimulation time $T_{\rm on}$ (black diamonds) of aDBS, where HF DBS was switched on, and its smoothed values (black dotted curves) are also shown. The LFP thresholds $Th_{\rm on} = Th_{\rm off} = 0.01$.

The mechanism of the beneficial effect of the interphase gap on stimulation-induced desynchronization as illustrated in Fig. S4A for pLDF seems to strongly relate to the impact of the shape of electrical pulses used for stimulation of the neuronal tissue as revealed in several experimental and modeling studies[9–11]. A monophasic pulse shape was found to have the strongest effect of neuronal activation, whereas the second recharging phase of the biphasic charge-balanced pulses counteracts the effect of the first pulse phase. In order to reduce this undesirable effect of the recharging phase, its amplitude can be reduced as

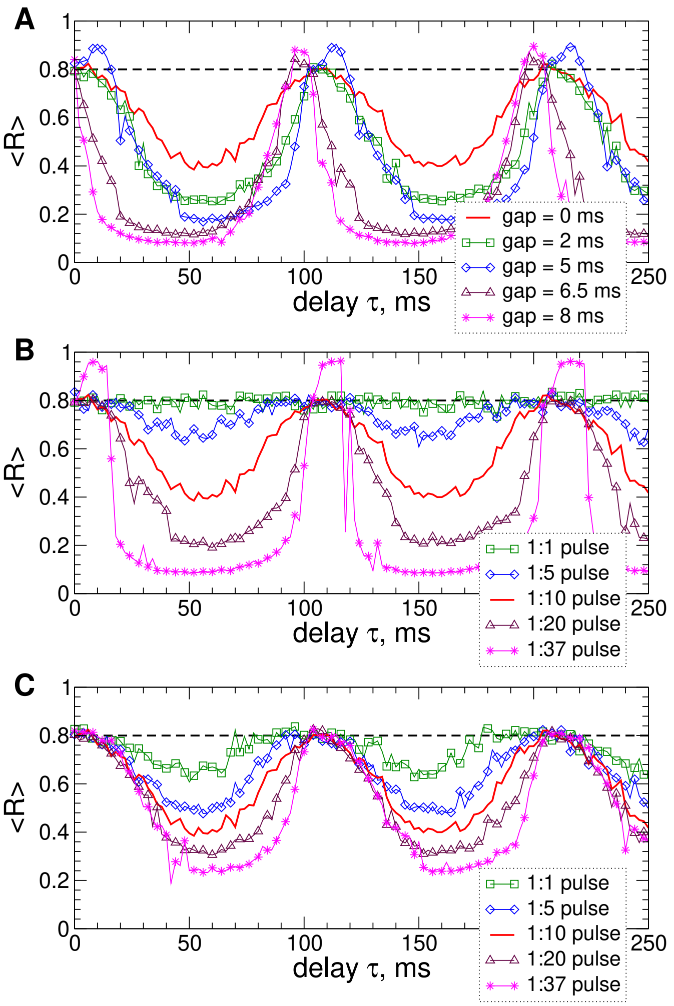

Figure S4. Suppression of synchronization in the STN-GPe neuronal populations (1) - (4) by cpLDF. Averaged order parameter $\langle R \rangle$ versus stimulation delay τ for the interphase gap and pulse shape as indicated in the legends. In plot (**A**) the pulse shape is fixed to the relation $1:10$ between the lengths of the first and the second recharging phases of the pulses as in Eq. (7). In plots (**B**) and (**C**) the gap width is fixed to $GW = 0$ ms. The horizontal dashed lines indicate the order parameter $\langle R \rangle$ of the stimulation-free STN neurons. Stimulation intensity (**A**), (**B**) $K = 10$, and (**C**) $K = 100$ for the pulse shape $1:1$, $K = 20$ for $1:5$, $K = 10$ for $1:10$, $K = 5$ for $1:20$, and $K = 2.7$ for $1:37$.

related to the amplitude of the first pulse phase, whereas its length is accordingly increased such that the charge-balancing property of the pulses is preserved. Such asymmetric biphasic charge-balanced pulses are typically used for HF DBS[12,13].

We illustrate how the pulse shape influences the extent of the stimulation-induced desynchronization in Fig. S4 B,C. The relation between the first and the second phases of the biphasic pulses [Fig. 2] is varied from $1:1$ (the lengths and amplitudes of the pulse phases are equal to each other) to $1:37$ (the recharging pulse phase is 37 times longer/weaker than the first pulse phase). In the latter case, for a fixed width of the 1st phase $PW = 0.2$ ms, the total pulse length is $0.2 + 37 * 0.2 = 7.6$ ms, which is slightly less than the interpulse interval $1000/130 \approx 7.69$ ms for 130 Hz HF pulse train. Increasing the difference in the amplitude of the 1st and 2nd pulse phases results in a certain "decoupling" of the stimulation and recharging pulse phases, which in turn reduces the counteracting effect of the recharging phase on the stimulation impact induced by the first pulse phase. At this the desynchronizing effect of pLDF stimulation can be enhanced [Fig. S4 B,C]. We illustrate it for two different setups, where the amplitude of either the second recharging phase or the first stimulation phase remains constant across different pulse shapes as shown in Figs. S4 B and S4 C, respectively. Based on the approach used for pulse modulation by the feedback signal [Fig. 2B], such stimulation setups can be realized either for constant stimulation intensity as in Fig. S4 B or by appropriately scaled stimulation intensity as in Fig. S4 C. For both stimulation conditions we observed that the desynchronizing outcome of

the stimulation can strongly be enhanced when the asymmetry of stimulation pulses increases.

The mentioned ”decoupling” between the first and the second phases of the pulses can further be enhanced together with an amelioration of the stimulation-induced desynchronization if an interphase time gap is introduced between pulse phases [Fig. S4A]. Note that for the 1 : 10 pulse shape and $PW = 0.2$ ms as in Fig. S4A, increasing the gap width beyond $GW \approx 5.49$ ms would destroy the charge-balancing property of the stimulation pulses (the recharging phase becomes too short) in this way turning into monophasic pulses for $GW \gtrsim 7.49$. As expected, the monophasic pulses can be most effective in inducing desynchronization by pLDF stimualtion, which we observe in Fig. S4A for $GW = 8$ ms. This makes the connection of the effects induced by the interphase gap and the pulse shape and explains the mechanisms of the observed effects in accordance with other modeling and experimental results[9–11].

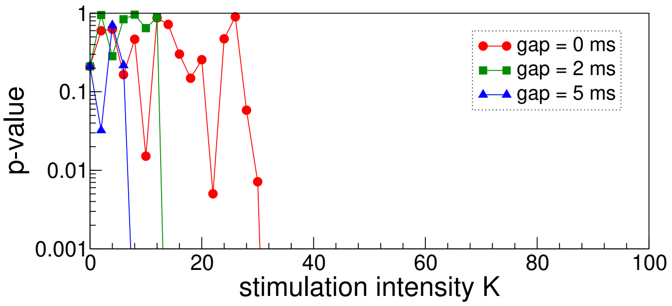

Figure S5. Statistical significance test of the difference between the length of the LFP bursts induced by cpLDF and apLDF illustrated in Fig. 10. The p-value of the Wilcoxon rank-sum test is depicted versus stimulation intensity K in the linear-log scale for the interphase gaps indicated in the legend. Other parameters as in Fig. 10.

References

1. Terman, D., Rubin, J. E., Yew, A. C. & Wilson, C. J. Activity patterns in a model for the subthalamopallidal network of the basal ganglia. *J. Neurosci.* **22**, 2963–2976 (2002).
2. Park, C., Worth, R. M. & Rubchinsky, L. L. Neural dynamics in parkinsonian brain: The boundary between synchronized and nonsynchronized dynamics. *Phys. Rev. E* **83**, 042901 (2011).
3. Hammond, C., Bergman, H. & Brown, P. Pathological synchronization in Parkinson’s disease: networks, models and treatments. *Trends Neurosci.* **30**, 357–364 (2007).
4. Benabid, A. L., Chabardes, S., Mitrofanis, J. & Pollak, P. Deep brain stimulation of the subthalamic nucleus for the treatment of Parkinson’s disease. *Lancet Neurol.* **8**, 67–81 (2009).
5. Kühn, A. A. *et al.* Pathological synchronisation in the subthalamic nucleus of patients with Parkinson’s disease relates to both bradykinesia and rigidity. *Exp. Neurol.* **215**, 380–387 (2009).
6. Adamchic, I. *et al.* Coordinated reset neuromodulation for Parkinson’s disease: Proof-of-concept study. *Mov. Disorders* **29**, 1679–1684 (2014).
7. Oswal, A. *et al.* Deep brain stimulation modulates synchrony within spatially and spectrally distinct resting state networks in Parkinson’s disease. *Brain* **139**, 1482–1496 (2016).
8. Neumann, W. J. *et al.* Deep brain recordings using an implanted pulse generator in parkinson’s disease. *Neuromodulation* **19**, 20–23 (2016).
9. Gorman, P. H. & Mortimer, J. T. The effect of stimulus parameters on the recruitment characteristics of direct nerve-stimulation. *IEEE Trans. Biomed. Eng.* **30**, 407–414 (1983).
10. Merrill, D. R., Bikson, M. & Jefferys, J. G. R. Electrical stimulation of excitable tissue: design of efficacious and safe protocols. *J. Neurosci. Methods* **141**, 171–198 (2005).
11. Hofmann, L., Ebert, M., Tass, P. A. & Hauptmann, C. Modified pulse shapes for effective neural stimulation. *Front. Neuroeng.* **4**, 9 (2011).

12. Butson, C. R. & McIntyre, C. C. Differences among implanted pulse generator waveforms cause variations in the neural response to deep brain stimulation. *Clin. Neurophysiol.* **118**, 1889–1894 (2007).

13. Coffey, R. J. Deep brain stimulation devices: A brief technical history and review. *Artif. Organs* **33**, 208–220 (2009).
